# Human Cases of Rift Valley Fever in South Africa, 2018

**DOI:** 10.1089/vbz.2018.2357

**Published:** 2018-11-28

**Authors:** Petrus Jansen van Vuren, Joe Kgaladi, Venessa Patharoo, Phumza Ohaebosim, Veerle Msimang, Babsy Nyokong, Janusz T. Paweska

**Affiliations:** ^1^ Centre for Emerging Zoonotic and Parasitic Diseases, National Institute for Communicable Diseases, National Health Laboratory Service, Sandringham, South Africa.; ^2^ Free State Department of Health, Bloemfontein, South Africa.

**Keywords:** Rift Valley fever, South Africa, IgM ELISA

## Abstract

Major Rift Valley fever (RVF) epidemics in South Africa occur at irregular intervals, usually spanning several decades, with human cases rarely reported in the absence of widespread outbreaks in livestock. This report describes four cases of RVF in farm workers associated with an isolated outbreak on a sheep farm in the Free State Province of South Africa, in 2018. In contrast to the last major RVF epidemic in South Africa in 2010–2011, where detection of human cases served as an alert for an ongoing outbreak in livestock, the current isolated outbreak was first detected in livestock, and human cases recognized following subsequent epidemiological investigation. This highlights the importance of early recognition of livestock cases in reducing risk and impact of a subsequent RVF epidemic in humans. People working with animals should be aware of transmission routes and take precautions to minimize risk of infection.

## Introduction

Rift Valley fever (RVF) is a mosquito-borne emerging disease in Africa, capable of causing large epidemics in livestock accompanied by cases in humans. Outbreaks in livestock are characterized by abortion storms and high mortality rates in young animals, while human infections are mostly mild with severe complications and death occurring only in a small proportion of affected individuals (Pepin et al. 2010). The RVF virus (RVFV) is a member of the *Phlebovirus* genus, family *Phenuiviridae* of the order Bunyavirales, transmitted by a wide range of mosquito vectors mainly from the *Aedes* and *Culex* genera, *Culicidae* family (Pepin et al. 2010, Maes et al. 2018).

Three major epidemics of RVF have been documented in South Africa during 1950–1951, 1974–1976, and 2010–2011. These primarily affected the Free State and Northern Cape provinces, but were widespread during the most recent two outbreaks involving also the Northern, Eastern, and Western Cape provinces (Archer et al. 2013). Smaller sporadic animal outbreaks with concurrent human cases have been documented in the periods between these major epidemics. The last major outbreak in South Africa in 2010–2011 was preceded by smaller isolated outbreaks in 2008 and 2009 in the north-east and north-west parts of the country (Archer et al. 2011).

In this study we report on human RVF cases associated with an isolated outbreak on a sheep farm in the Free State Province of South Africa at the end of the 2017–2018 mosquito season (OIE 2018). The increased rainfall in South Africa during 2017–2018, following a drought period, and prediction for more sustained rain over the coming years serve as a reliable risk indicator of potential RVF outbreaks. Although limited, this first recognized RVF outbreak since 2011 must be viewed as a warning for the possibility of an extensive epidemic in the ensuing years. Farmers in the area have been advised by the Department of Agriculture, Forestry and Fisheries, Animal Production and Health of South Africa to vaccinate their livestock, in an effort to prevent larger outbreaks during the next rainy season.

## Materials and Methods

Six of 22 individuals residing or working on the RVF-affected sheep farm (OIE 2018) were identified as having recently experienced symptoms compatible with RVFV infection, during the initial epidemiological investigation. The six individuals were interviewed using a standard RVF case investigation form pertaining to questions on demographic details and symptoms and designed to evaluate their exposure. Clotted blood samples were collected at a single time point from these six individuals and submitted to the National Institute for Communicable Diseases for RVF laboratory testing. Laboratory testing on serum consisted of: RVF real-time RT-PCR (Drosten et al. 2002), hemagglutination inhibition assay (HAI) (Swanepoel et al. 1986), RVF inhibition ELISA (Paweska et al. 2005a), and RVF IgM ELISA (Paweska et al. 2005b) as described previously. RNA was extracted from serum using QIAamp Viral RNA Kit (Qiagen, Germany) following the manufacturer's instructions.

## Results and Discussion

On May 16, 2018, an outbreak of RVF was reported in sheep on a farm in the Jacobsdal area in the Free State Province of South Africa by the Department of Agriculture, Forestry and Fisheries, Animal Production and Health of South Africa (OIE 2018). The event, which started on April 28, 2018, resulted in a total of 250 fatalities/abortions from a flock of 600 sheep on a single farm. Following the announcement of the outbreak, health officials from the Free State Department of Health conducted an investigation on the affected farm on May 21, 2018, to determine the possible occurrence of human cases. A total of 6 of 22 people on the farm were identified as having experienced RVF compatible symptoms, during this first visit. Laboratory results and epidemiological data are summarized in Table 1.

**Table 1.** Summary of Laboratory Findings and Epidemiological Data Collected from Six Sampled Individuals

*Case no.*	*Age, years*	*Sex*	*Exposure history*^a^	*Symptoms*	*RVF qRT-PCR*^b^	*RVF HAI titer*	*RVF inhibition ELISA (PI value)*^c^	*RVF IgM ELISA (PP value)*^d^
SA342/18	38	M	Slaughtering, disposal of carcasses, consumption of meat	Headache, muscle pain, history of fever	Negative	Negative	Negative (2.05 PI)	Negative (0.22 PP)
SA343/18	35	F	Disposal of carcasses, consumption of raw meat, cook meat	Headache, muscle pain, rigors, nausea	Negative	1:40	Positive (85.99 PI)	Positive (127 PP)
SA344/18	38	M	Slaughtering, disposal of carcasses/aborted fetuses, consumption of meat	Headache, muscle pain	Negative	1:640	Positive (97.16 PI)	Positive (154 PP)
SA345/18	21	M	Slaughtering, consumption of meat, autopsy on carcasses	Headache, muscle pain, rigors, history of fever	Negative	1:640	Positive (96.40 PI)	Positive (151 PP)
SA346/18	34	M	Slaughtering, disposal of carcasses, consumption of meat	Headache, muscle pain, body ache, history of fever	Negative	1:320	Positive (92.98 PI)	Positive (111 PP)
SA347/18	45	M	Slaughtering, autopsy, disposal of carcasses/aborted fetuses	Headache, muscle pain	Negative	Negative	Negative (2.17 PI)	Negative (0 PP)

^a^ All individuals were estimated to have been exposed for 3 weeks (± 23 days) before sampling, deduced from date of animal cases and date of sampling.

^b^ RT-PCR performed on RNA extracted from serum only.

^c^ PI value = inhibition ELISA percentage inhibition relative to a negative control (cutoff value 38.6 PI). This ELISA detects total antibodies (IgM and IgG).

^d^ PP value = capture IgM ELISA percentage positivity value relative to a positive control (cutoff value 7.1 PP).

HAI, hemagglutination inhibition assay; PI, percentage inhibition; RVF, Rift Valley fever.

All six individuals were interviewed and sampled on May 21, 2018, ∼3 weeks after the documented animal cases. All of them indicated time of exposure by the end of April, beginning of May 2018, which matched with the report on animal cases. The time lapse between the animal cases, announcement of the outbreak and the public health response, made it difficult for the persons to recall specific dates of symptom onset. However, all individuals estimated onset to be around middle May 2018. None of the symptomatic individuals required hospitalization. All six workers had engaged in activities, that is, slaughter, disposal of infected carcasses, or aborted lambs, which are known to constitute a high risk for RVFV infection (Archer et al. 2013). None had reported drinking of raw unpasteurized milk, but some had consumed meat of the infected animals.

The absence of RVFV RNA in the serum of all tested people is compatible with the 7-day lapse among estimated date of onset, date of collection, and the documented transient viremia in RVFV infected humans (Pepin et al. 2010). Four of the six individuals were confirmed to be recently infected with RVFV through detection of serum specific antibodies (IgM detected by capture ELISA and total antibodies detected by Inhibition ELISA and HAI). No evidence of RVFV infection could be found in two of the sampled individuals, even though they reported similar exposure history and symptoms.

The detection of these cases reaffirms the risk for RVFV infection in individuals participating in slaughtering and handling of possibly infected animals and their tissues in RVF endemic areas. Averting the negative socioeconomic impact of RVF can be achieved through vaccination of livestock, preferably in early summer since historically most South African outbreaks have occurred in late summer to autumn (Archer et al. 2013). In South Africa, three livestock RVF vaccines are commercially available (www.obpvaccines.co.za/products), including an inactivated formulation and two live attenuated vaccine strains (Smithburn and Clone-13). Choice of vaccine type is mostly dependent on age and pregnancy status of animals to be vaccinated. Further protection of human health can be achieved through education of individuals participating in high-risk activities and use of simple personal protective equipment, such as heavy-duty rubber gloves and full-face protection to protect exposed mucosal surfaces.

The interepidemic maintenance of RVFV in South Africa is not well understood, and the isolated nature of the 2018 outbreak might indicate that the virus is maintained in specific foci which feed an expansion and perpetuation following sustained rains, to result in larger epidemics. No RVF cases have been reported from other farms in the area at the time of writing, but retrospective surveillance in livestock and humans in the area could confirm whether this was truly a single farm event. The outbreak reported here occurred at the start of winter in South Africa, when conditions are not conducive to breeding of large mosquito populations or extensive onward arbovirus transmission. The detection of this isolated outbreak might be considered as a warning for more widespread outbreaks in the ensuing rainy season, similar to the events leading up to the 2010–2011 outbreak, should farmers not heed the advice to ensure vaccination of their livestock.
